# Genetic risk profiling for prediction of type 2 diabetes

**DOI:** 10.1371/currents.RRN1208

**Published:** 2011-01-11

**Authors:** Raluca Mihaescu, James Meigs, Eric Sijbrands, A. Cecile Janssens

**Affiliations:** ^*^Erasmus University Medical Center Rotterdam; ^†^Massachusetts General Hospital and ^‡^Dept. of Internal Medicine, Erasmus MC, Rotterdam, The Netherlands

## Abstract

Type 2 diabetes (T2D) is a common disease caused by a complex interplay between many genetic and environmental factors. Candidate gene studies and recent collaborative genome-wide association efforts revealed at least 38 common single nucleotide polymorphisms (SNPs) associated with increased risk of T2D. Genetic testing of multiple SNPs is considered a potentially useful tool for early detection of individuals at high diabetes risk leading to improved targeting of preventive interventions.

**Clinical Scenario**

Both a population-based approach and a targeted high-risk approach are recommended as strategies for prevention of T2D. Several recent guidelines advocate screening for individuals at risk to develop T2D followed by blood glucose measurements to detect individuals with impaired fasting glucose (IFG) or impaired glucose tolerance (IGT).[1] Genetic testing of a panel of SNPs may be useful in detecting such groups of high-risk individuals in whom screening for T2D could be optimized.  

**Test Description**

Genetic susceptibility testing for T2D is currently offered by several commercial companies that use genome-wide scans to deliver information about risk for many common complex diseases (see **Table 1**). For example, deCODEme offers predictions for 50 complex diseases and non-disease phenotypes that vary from breast cancer, atrial fibrillation, T2D or psoriasis, to eye color and bitter taste perception.[2] Tests are available for purchase directly to the individual consumers, or through the request from a physician (see **Table 2**). 

Direct-to-consumer risk companies sell risk profiles that differ in the number of genetic markers included and in the exact SNPs used. For example, deCODEme uses 21 SNPs from the genome-wide scan to calculate the risk of T2D for individuals with European descent, 9 SNPs for East Asians and 2 SNPsfor African Americans.[3] A test based on the same markers is also available as a separate T2D profile.[4] Pathway Genomics offers also separate tests for individuals of African, Asian and Caucasian origin,[5] 23andMe uses 9 SNPs to determine the risk of developing T2D,[6] Navigenics tests18 SNPs,[7] and GeneticHealth, an UK based company, calculates the risk for obesity, diabetes and weight loss using the same 7 SNPs.[8]

Genetic risks are calculated on the basis of literature data. The companies take an average risk from some epidemiological study and multiply this with the odds ratios from published meta-analyses or large scale genome-wide association studies.[9] Importantly, the companies do not use information about clinical risk factors when calculating the risk of disease. When available, some companies use sex, ethnicity and age matched population risks to depart from.

**Table 1. Direct-to-consumer companies that sell genetic tests for Type 2 Diabetes risk**

**Table:** 

**Company name**	**Predict multiple diseases at the same time**	**URL**
deCODEme	Yes No	http://www.decodeme.com http://www.decodediagnostics.com
23andMe	Yes	https://www.23andme.com
Navigenics	Yes	http://www.navigenics.com
Pathway Genomics	Yes	http://www.pathway.com
GeneticHealth	No	http://www.genetic-health.co.uk
GHC Genetics	Yes	http://www.genscan.com/en/
CyGene Direct	Yes	http://www.cygenedirect.com

**Table 2. Direct-to-consumer companies**

**Table:** 

**Company name**	**Delivery model**	**Accreditation**
deCODEme	DTC	CLIA
23andMe	DTC	CLIA
Navigenics	Through physician or corporate wellness program	CLIA
Pathway Genomics	Through physician	CLIA
GeneticHealth	DTC	Not mentioned on website
GHC Genetics	DTC and through physician	ISO 9001:2001 ISO 27001:2006
CyGene Direct	DTC	CLIA

****

Legend **Table 2**: CLIA, Clinical Laboratory Improvement Amendments of 1988; DTC, direct-to-consumer.

**Public Health Importance**

T2D is a metabolic disorder characterized by hyperglycemia, insulin resistance and relative insulin deficiency. Diabetes is a leading cause of blindness, renal failure and limb amputation, and a major risk factor for cardiovascular morbidity and mortality.[10] It is estimated that approximately 285 million people worldwide will have diabetes in 2010. This number is expected to increase by more than 50% in the next 20 years if no preventive strategies are implemented.[11] Diabetes is responsible for almost four million deaths worldwide in the 20-79 age group in 2010, representing 6.8% of global all-cause mortality in this age group.[11]

Preventive interventions for T2D, including medication, weight loss and increased physical activity, can slow or even reverse the disease process.[12] For example, the United States Diabetes Prevention Program trial investigated the efficacy of intensive lifestyle interventions or metformin treatment compared to standard lifestyle recommendations.[13] Lifestyle intervention resulted in 58% T2D risk reduction compared to the placebo arm, at 2.8 years of follow-up. For the same follow-up, metformin resulted in 31% T2D risk reduction.[13] Genetic tests are claimed by the DTC companies to improve risk prediction and increase adherence to preventive interventions (e.g., “Knowledge is self-empowering and it can motivate you towards taking steps that reduce other risk factors, which have been found to contribute to your genetic predisposition risk”[14]), thus helping to improve outcomes and reduce the costs and burden of disease for society (e.g., “The conditions included in Navigenics’ analysis are those that are clinically actionable and those that contribute to the major burden of disease in the United States, such as myocardial infarction, cancer, and type 2 diabetes.”[15])

**Published Reviews, Recommendations and Guidelines**

**Systematic evidence reviews**

None identified.

**Recommendations by independent group**

None identified.

**Guidelines by professional groups**

None identified.

A European multidisciplinary consortium developed an evidence-based guideline for the prevention of T2D. The consortium advocates the use of clinical risk scores as primary screening tools to identify high-risk groups in whom T2D screening may be targeted more efficiently. One such example is the Finnish risk test (FINDRISC) that provides ten-year risks to develop T2D. The FINDRISC score contains eight items: age, BMI, waist circumference, antihypertensive medication, history of elevated blood glucose, daily physical activity and daily intake of fruits or vegetables. In the context of targeted screening, the guideline includes the followingrecommendation about genetic testing**:** “despite the encouraging progress in our understanding of the genetic basis of T2DM, it is too early to use genetic information as a tool for targeting preventive efforts”.[1] No other guidelines provide recommendations for or against the use of genetic testing for screening, prevention or treatment of T2D. 

**Evidence Overview**

***Analytic Validity*: **Test accuracy and reliability in identifying multiple SNPs (analytic sensitivity and specificity).

Navigenics reports an analytic accuracy of 99%,[15] deCODEme does not provide a measure of accuracy but describes the methods used to ensure good analytic validity,[16] 23andMe does not disclose the methods used,[17] and the same applies to Pathway Genomics.[18] CyGene Direct briefly mentions the methods used to ensure good analytic validity.[19] No information was available on the analytic validity of commercial tests for GeneticHealth and GHC Genetics.

Direct-to-consumer genetic testing services are not clearly regulated by governmental agencies. Their services may bypass healthcare providers who are typically responsible for appropriate ordering of lab tests and for discussing with patients the implications of test results. Not all companies explicitly report the analytic and clinical performance characteristics of their test systems. Following a recent Government Accountability Office investigation of companies providing direct-to-consumer genetic tests, the US Food and Drug Administration is considering premarket review of some laboratory-derived tests that pose higher clinical risks, assuring that the tests are evaluatedfor analytical and clinical validity.[20]

***Clinical Validity*:** Clinical validity refers to test accuracy and reliability in predicting risk of T2D (discrimination and calibration).

Discrimination shows how well the model can distinguish between individuals with and without disease. A commonly used measure of discrimination is the area under the receiver operating characteristic curve (AUC). AUC can vary from 0.5 (equal to tossing a coin) to 1 (perfect discrimination). AUC indicates the probability that, on average, an individual with the disease will be assigned a higher predicted risk than an individual without the disease. Calibration indicates how close the risks predicted by the model are to the actual observed risks. The Hosmer-Lemeshow (H-L) chi-square test is a commonly used summary measure of calibration. The H-L test compares the observed and predicted number of patients within specified risk groups, usually deciles of risk. 

In most empirical studies, the genetic risk scores had lower discriminative accuracy than the clinical risk factors.[21] [22] [23] Furthermore, addition of genetic factors to the clinical risk factors either did not change or only marginally improved the AUC beyond the clinical risk models. Like companies, all studies used multiplicative models or additive genetic effects,[24] [25] [26] but whether this is correct has not been demonstrated. Besides, none of these studies investigated the same panel of SNPs as the companies do. Disagreement between results for identical DNA samples sent to 4 different companies reflects the use of different sets of markers to predict risk of disease and the use of different average risks to depart from.[20] [27] **Table 3** presents an overview of the published studies conducted on T2D risk so far, mostly in European populations. **Table 4** shows the SNPs included in genetic risk scores in the studies summarized in **Table 1** and the SNPs used by three commercial companies to predict T2D risk. The other companies do not specify on their websites which SNPs they use for T2D risk prediction. For most companies, algorithms or criteria for interpreting SNP results are not made clear to the consumer. Even when this information is made available,[24] [25] [26] it is sometimes difficult to know which effect sizes and genotype frequencies are used to calculate a composite risk.[28]

**Table 3.** Genetic risk prediction studies in type 2 diabetes 

**Table:** 

**Study reference**	**Design**	**Sample size**	**Clinical risk factors**	**No of SNPs **	**AUC clinical** **(95%CI)**	**AUC genetic** **(95% CI)**	**AUC both ** **(95% CI)**
**European**							
Weedon et al.[29]	Case-control	2409 T2D cases / 3668 controls	NA	3	NA	0.58	NA
Lyssenko et al.[30] [31]	Prospective cohort	2293	BMI, FPG	3	0.68 (0.63 to 0.73)	NA	0.68 (0.63 to 0.73)
Vaxillaire et al.[32]	Prospective cohort	3877	Age, sex, BMI	3	0.82	0.56	0.83
Cauchi et al.[33]	Case-control	4232 T2D cases / 4595 controls	Age, sex, BMI	15	NA	NA	0.86
van Hoek et al.[34]	Prospective cohort	6544	Age, sex, BMI	18	0.66 (0.63 to 0.68)	0.60 (0.57 to 0.63)	0.68 (0.66 to 0.71)
Lango et al.[35]	Case-control	2309 T2D cases / 2598 controls	Age, sex, BMI	18	0.78	0.60	0.80
Lyssenko et al.[36]	Prospective cohorts	1) Malmö study: 16,061 2) Botnia study: 2770	Age, sex, BMI, FH, BP, TG, FPG (in Malmö study), plus HDL and waist circumference (in Botnia study	11	1) 0.743 2) 0.786	1) 0.626 2) 0.682	1) 0.753 2) 0.801
Meigs et al.[37]	Prospective cohort	2377	Age, sex, BMI, FH, FPG, SBP, HDL, TG	18	0.900 (0.880 to 0.919)	0.581 (0.546 to 0.617) (adjusted for sex)	0.901 (0.881 to 0.920)
Balkau et al.[38]	Prospective cohort	1) Men: 1863 2) Women: 1954	Current smoker (in men), waist circumference, HT, FPG, GGT, and FH and BMI (in women)	2	1)0.850 2) 0.917	NA	1) 0.851 2) 0.912
Lin et al.[39]	Cross-sectional	356 T2D cases / 5004 controls	Age, sex, FH, physical activity, triacylglycerol/HDL ratio, waist-hip ratio	15	0.86	0.59	0.87
Sparso et al.[40]	Case-control	4093 T2D cases / 5302 controls	Age, sex, BMI	19	0.92	0.60	0.93
Schulze et al.[41]	Prospective case-cohort	579 T2D cases / 1962 controls	Age, waist circumference, height, history of HT, physical activity, smoking, consumption of red meat, whole-grain bread, coffee and alcohol, glucose, HbA1c, TG, HDL, GGT, ALT, hs-CRP	20	0.90 (0.89 to 0.92)	NA	0.90 (0.89 to 0.91)
Cornelis et al.[42]	Nested case-control	2768 T2D cases / 3447 controls	Age, sex, BMI, FH, smoking, alcohol intake, physical activity	10	0.78 (0.77 to 0.79)	NA	0.79 (0.78 to 0.80)
Talmud et al.[43]	Prospective cohort	5135	1) Cambridge T2D risk score: age, sex, BMI, drug treatment, FH, smoking status 2) Framingham offspring T2D risk score: age, sex, BMI, parental history of T2D, HDL, TG, FPG	20	1) 0.72 (0.69 to 0.76) 2) 0.78 (0.75 to 0.82)	0.55 (0.51 to 0.59)	1) 0.73 (0.69 to 0.76) 2) 0.78 (0.74 to 0.81)
Fontaine-Bisson et al.[44]	Cross-sectional	1327 T2D cases / 1424 controls	Age, sex	17	NA	NA	0.591
Wang et al.[45]	Cross-sectional	518 T2D cases / 6714 controls	1) Finnish Diabetes Risk Score (FINDRISC): age, BMI, waist circumference, antihypertensive medication, physical activity, previously known high glucose, FH, diet 2) FINDRISC, TG, HDL, adiponectin, ALT	19	1) 0.727 (0.705 to 0.749) 2) 0.772 (0.752 to 0.793)	0.552 (0.526 to 0.578)	1) 0.730 (0.708 to 0.753) 2) 0.772 (0.751 to 0.793)
de Miguel-Yanes et al.[46]	Prospective cohort	3471 (11 358 person-observations)	1) Sex, FH, BMI, FPG, SBP, HDL, TG; stratified by age 2) Age, sex, FH, BMI, FPG, SBP, HDL, TG	40	1) <50 yrs: 0.908 (0.884 to 0.932) ≥50 yrs: 0.883 (0.863 to 0.903) 2) 0.903 (0.889 to 0.917)	1) <50 yrs: 0.657 (0.611 to 0.703) ≥50 y: 0.590 (0.557 to 0.623) 2) 0.606 (0.579 to 0.632) (adjusted for sex)	1) <50 yrs: 0.911 (0.887 to 0.935) ≥50 yrs: 0.884 (0.865 to 0.904) 2) 0.906 (0.892 to 0.920)
**Asian** ** **							
Miyake et al.[47]	Case-control	2316 cases / 2370 controls	Age, sex, BMI	11	0.68	0.63	0.72
Hu et al.[48]	Case-control	1849 T2D cases / 1785 controls	Age, sex, BMI	11	0.614 (0.595 to 0.632)	0.621 (0.604 to 0.639)	0.668 (0.650 to 0.685)

Legend **Table 3**: ALT, alanine aminotransferase; BMI, body mass index; BP, blood pressure; FH, family history of T2D; FPG, fasting plasma glucose; GGT, γ-glutamyltransferase; HDL, high-density lipoprotein cholesterol; hs-CRP, high sensitivity c-reactive protein; HT, hypertension; NA, not available; SBP, systolic blood pressure; SNPs, single nucleotide polymorphisms; TG, triglycerides. 

Search strategy: We performed a search in PubMed and HuGE Navigator to identify relevant studies, scanned the reference lists from the retrieved articles to identify additional studies, and further used Web of Science to identify studies that cited the selected articles. The specific queries used are provided under the heading **Links**.

**Table 4.** Single nucleotide polymorphisms tested in risk prediction studies and used by commercial companies to predict type 2 diabetes risk

**Table 4A**

**Table:** 

** **		**Direct-to-Consumer Companies**				
**Locus**	**SNPs**	**deCODEme (European ancestry)**	**deCODEme (East Asian ancestry)**	**deCODEme (African ancestry)**	**23andme**	**Navigenics**
*TCF7L2*	rs7903146	X	X	X	X	X^1^
*TCF7L2*	rs7901695^2^	-	-	-	-	-
*TCF7L2*	rs12255372^2^	-	-	-	-	-
*PPARG*	rs1801282	X	-	-	X	X
*CDKN2A/B*	rs10811661	X	X^1^	-	X^1^	X
*CDKN2A/B*	rs564398^ 4^	-	-	-	-	-
*CDKN2A/B*	rs1412829^ 4^	-	-	-	-	-
*KCNJ11*	rs5219	X^1^	X^1^	-	X	X^1^
*IGF2BP2*	rs4402960	X	X	-	X	X
*SLC30A8*	rs13266634	X	X	-	X	X
*HHEX-IDE-KIF11*	rs1111875	X	X	-	X	X
*HHEX-IDE-KIF11*	rs7923837^ 2^	-	-	-	-	-
*HHEX-IDE-KIF11*	rs10748582^2^	-	-	-	-	-
*WFS1*	rs10010131	X	-	-	X^1^	X
*NOTCH2/ADAM30*	rs10923931	X^1^	-	-	-	X
*JAZF1*	rs864745	X	-	-	-	X
*THADA*	rs7578597	X	-	-	-	-
*TSPAN8/LGR5*	rs7961581	X	-	-	-	X
*TCF2/HNF1B*	rs4430796	X	X	X	-	X
*TCF2/HNF1B*	rs757210^ 2^	-	-	-	-	-
*TCF2/HNF1B*	rs7501939^ 2^	-	-	-	-	-
*CDKAL1*	rs7756992	X	X	-	-	X
*CDKAL1*	rs7754840 ^2^	-	-	-	-	-
*CDKAL1*	rs10946398^2^	-	-	-	-	-
*CDKAL1*	rs4712523^ 2^	-	-	-	X	-
*CDKAL1*	rs17036101^4^	-	-	-	-	-
*KCNQ1*	rs2237892	X	X	-	-	X^1^
*KCNQ1*	rs2237895^ 4^	-	-	-	-	-
*KCNQ1*	rs231362^ 4^	-	-	-	-	-
*MTNR1B*	rs10830963	X	-	-	-	-
*GCK*	rs4607517	X	-	-	-	-
*GCKR*	rs780094	X	-	-	-	-
*DGKB/TMEM195*	rs2191349	X^1^	-	-	-	-
*ADCY5*	rs2877716	X	-	-	-	-
*PROX1*	rs340874	X	-	-	-	-
*FTO*	rs8050136	-	-	-	-	X
*ADAMTS9*	rs4607103	-	-	-	-	X
*CDC123/CAMK1D*	rs12779790	-	-	-	-	-
*CDC123/CAMK1D*	rs11257622^2^	-	-	-	-	-
*VEGFA*	rs9472138	-	-	-	-	-
*DCD*	rs1153188	-	-	-	-	-
*BCL11A*	rs10490072	-	-	-	-	-
*BCL11A*	rs243021^ 4^	-	-	-	-	-
*LOC387761*	rs7480010	-	-	-	-	-
*CAPN10*	rs3792267	-	-	-	-	-
*CAPN10*	rs2975760^ 3^	-	-	-	-	-
*EXT2*	rs3740878	-	-	-	-	-
*IL6*	rs1800795	-	-	-	-	-
*chr11.41871942*	rs9300039	-	-	-	-	X
*LOC441171*	rs9494266	-	-	-	-	X
*CAMTA1*	rs1193179	-	-	-	-	-
*CXCR4*	rs932206	-	-	-	-	-
*INS*	rs689	-	-	-	-	-
*KCTD12*	rs2876711	-	-	-	-	-
*LDLR*	rs6413504	-	-	-	-	-
*LOC646279*	rs1256517	-	-	-	-	-
*MMP26*	rs2499953	-	-	-	-	-
*NGN3*	rs10823406	-	-	-	-	-
*HNF1A*	rs1800574	-	-	-	-	-
*CENTD2*	rs1552224	-	-	-	-	-
*HCCA2*	rs2334499	-	-	-	-	-
*HMGA2*	rs1531343	-	-	-	-	-
*KIAA1486*	rs7578326	-	-	-	-	-
*KLF14*	rs972283	-	-	-	-	-
*OASL/TCF1*	rs7957197	-	-	-	-	-
*PRC1*	rs8042680	-	-	-	-	-
*RBMS1/ITGB6*	rs7593730	-	-	-	-	-
*TLE4*	rs13292136	-	-	-	-	-
*TP53INP1*	rs896854	-	-	-	-	-
*ZBED3*	rs4457053	-	-	-	-	-
*ZFAND6*	rs11634397	-	-	-	-	-

**Table 4B**

**Table:** 

** **		**Variants Evaluated**																			
** **		**Published Studies**																			
**Locus**	**SNPs**	29	30, 31	32	33	34	35	36	37	47	38	48	39	40	41	42	43	44	45	46	
*TCF7L2*	rs7903146	X	-	X	X	X	X	X	X	X	X	-	-	X	X	-	-	X	X	X	
*TCF7L2*	rs7901695^ e^	-	-	-	-	-	-	-	-	-	-	-	X	-	-	-	X	-	-	-	
*TCF7L2*	rs12255372^e^	-	-	-	-	-	-	-	-	-	-	-	-	-	-	X	-	-	-	-	
*PPARG*	rs1801282	X	X	-	-	X	X	X	X	X	-	X	X	X	X	X	X	X	X	X	
*CDKN2A/B*	rs10811661	-	-	-	X	X	X	-	X	X	-	X	X	X	X	X	X	X	X	X	
*CDKN2A/B*	rs564398^ g^	-	-	-	-	-	X	-	-	-	-	-	-	-	-	X	-	-	-	-	
*CDKN2A/B*	rs1412829^ g^	-	-	-	-	X	-	-	-	-	-	-	-	-	-	-	-	-	-	-	
*KCNJ11*	rs5219	X	-	-	-	X	X	X	X	X	-	X	X^d^	X^d^	X	X	X^d^	X	X	X^d^	
*IGF2BP2*	rs4402960	-	-	-	X	X	X	X	X^d^	X^d^	-	X^d^	X	X	X	X	X	-	X	X^d^	
*SLC30A8*	rs13266634	-	-	-	X	X	X	X	X	X^d^	-	X	-	X	X	X	X	X	X	X	
*HHEX-IDE-KIF11*	rs1111875	-	-	-	-	X	X	X	X	-	-	-	X	X	X	X	X	X	X	X	
*HHEX-IDE-KIF11*	rs7923837^ e^	-	-	-	X	-	-	-	-	X	-	-	-	-	-	-	-	X	-	-	
*HHEX-IDE-KIF11*	rs10748582^e^	-	-	-	-	-	-	-	-	-	-	X	-	-	-	-	-	-	-	-	
*WFS1*	rs10010131	-	-	-	-	X^d^	X	X	-	-	-	X	X	X	X	X	-	X	X	X	
*NOTCH2/ADAM30*	rs10923931	-	-	-	-	X^d^	X^d^	X	X	-	-	-	X	X	X	-	X	X	X	X	
*JAZF1*	rs864745	-	-	-	-	X^d^	X	X	X	-	-	-	X	X	X	-	X	-	X	X	
*THADA*	rs7578597	-	-	-	-	X	X	-	X	-	-	-	X	X	X	-	X	X	X	X	
*TSPAN8/LGR5*	rs7961581	-	-	-	-	X^d^	X	-	X	-	-	-	X	X	X	-	X	X	X	X	
*TCF2/HNF1B*	rs4430796	-	-	-	-	X	-	-	-	X	-	X	-	-	X	-	X	X	-	-	
*TCF2/HNF1B*	rs757210^ e^	-	-	-	-	-	X	-	-	-	-	-	-	-	-	-	-	-	-	X	
*TCF2/HNF1B*	rs7501939^ e^	-	-	-	-	-	-	-	-	-	-	-	-	X	-	-	-	-	-	-	
*CDKAL1*	rs7756992	-	-	-	X	-	-	-	-	-	-	X	-	X	-	X	-	-	-	-	
*CDKAL1*	rs7754840 ^e^	-	-	-	-	X	-	X	X	X	-	-	-	-	X	-	-	-	X	X	
*CDKAL1*	rs10946398^e^	-	-	-	-	-	X	-	-	-	-	-	X	-	-	-	-	-	-	-	
*CDKAL1*	rs4712523^ e^	-	-	-	-	-	-	-	-	-	-	-	-	-	-	-	-	-	-	-	
*CDKAL1*	rs17036101^g^	-		-	-	-	-	-	-	-	-	-	-	-	-	-	X	-	-	-	
*KCNQ1*	rs2237892	-	-	-	-	-	-	-	-	X	-	X		-	-	-	-	-	X^d^	X	
*KCNQ1*	rs2237895^ g^	-	-	-	-	-	-	-	-	-	-	-	-	X	-	-	-	-	-	-	
*KCNQ1*	rs231362^ g^	-	-	-	-	-	-	-	-	-	-	-	-	-	-	-	-	-	-	X	
*MTNR1B*	rs10830963	-	-	-	-	-	-	-	-	-	-	-	-	X	-	-	-	-	X	X	
*GCK*	rs4607517	-	-	X^d^	-	-	-	-	-	-	-	-	-	-	-	-	-	-	-	X	
*GCKR*	rs780094	-	-	-	-	-	-	-	-	X	-	-	-	-	-	-	-	-	-	X	
*DGKB/TMEM195*	rs2191349	-	-	-	-	-	-	-	-	-	-	-	-	-	-	-	-	-	-	X	
*ADCY5*	rs2877716	-	-	-	-	-	-	-	-	-	-	-	-	-	-	-	-	-	-	X^d^	
*PROX1*	rs340874	-	-	-	-	-	-	-	-	-	-	-	-	-	-	-	-	-	-	X	
*FTO*	rs8050136	-	-	-	-	X	X	X^d^	-	-	-	X	X	X	X	-	X	-	X^d^	X^d^	
*ADAMTS9*	rs4607103	-	-	-	-	X^d^	X	-	X	-	-	-	X	X	X	-	X	-	X	X	
*CDC123/CAMK1D*	rs12779790	-	-	-	-	-	X	-	X	-	-	-	X	X	X	-	X	X	X	X	
*CDC123/CAMK1D*	rs11257622^e^	-	-	-	-	X	-	-	-	-	-	-	-	-	-	-	-	-	-	-	
*VEGFA*	rs9472138	-	-	-	-	-	-	-	X	-	-	-	-	-	X	-	X	X	-	X	
*DCD*	rs1153188	-	-	-	-	-	-	-	X	-	-	-	-	-	X	-	-	X	-	X	
*BCL11A*	rs10490072	-	-	-	-	-	-	-	X	-	-	-	-	-	X	-	X	-	-	-	
*BCL11A*	rs243021^ g^	-	-	-	-	-	-	-	-	-	-	-	-	-	-	-	-	-	-	X	
*LOC387761*	rs7480010	-	-	-	X	-	-	-	-	-	-	-	-	-	-	-	-	X	X	-	
*CAPN10*	rs3792267	-	X	-	-	-	-	-	-	-	-	-	-	-	-	-	X	-	-	-	
*CAPN10*	rs2975760^ f^	-	X	-	-	-	-	-	-	-	-	-	-	-	-	-	-	-	-	-	
*EXT2*	rs3740878	-	-	-	X	-	-	-	-	-	-	-	-	-	-	-	-	X^d^	-	-	
*IL6*	rs1800795	-	-	X	-	-	-	-	-	-	X	-	-	-	-	-	-	-	-	-	
*chr11.41871942*	rs9300039	-	-	-	-	-	-	-	-	-	-	-	-	-	-	-	-	-	-	-	
*LOC441171*	rs9494266	-	-	-	-	-	-	-	-	-	-	-	-	-	-	-	-	-	-	-	
*CAMTA1*	rs1193179	-	-	-	X	-	-	-	-	-	-	-	-	-	-	-	-	-	-	-	
*CXCR4*	rs932206	-	-	-	X	-	-	-	-	-	-	-	-	-	-	-	-	-	-	-	
*INS*	rs689	-	-	-	-	-	-	-	X	-	-	-	-	-	-	-	-	-	-	-	
*KCTD12*	rs2876711	-	-	-	X	-	-	-	-	-	-	-	-	-	-	-	-	-	-	-	
*LDLR*	rs6413504	-	-	-	X	-	-	-	-	-	-	-	-	-	-	-	-	-	-	-	
*LOC646279*	rs1256517	-	-	-	X	-	-	-	-	-	-	-	-	-	-	-	-	-	-	-	
*MMP26*	rs2499953	-	-	-	X	-	-	-	-	-	-	-	-	-	-	-	-	-	-	-	
*NGN3*	rs10823406	-	-	-	X	-	-	-	-	-	-	-	-	-	-	-	-	-	-	-	
*HNF1A*	rs1800574	-	-	-	-	-	-	-	-	-	-	-	-	-	-	-	X	-	-	-	
*CENTD2*	rs1552224	-	-	-	-	-	-	-	-	-	-	-	-	-	-	-	-	-	-	X	
*HCCA2*	rs2334499	-	-	-	-	-	-	-	-	-	-	-	-	-	-	-	-	-	-	X	
*HMGA2*	rs1531343	-	-	-	-	-	-	-	-	-	-	-	-	-	-	-	-	-	-	X	
*KIAA1486*	rs7578326	-	-	-	-	-	-	-	-	-	-	-	-	-	-	-	-	-	-	X	
*KLF14*	rs972283	-	-	-	-	-	-	-	-	-	-	-	-	-	-	-	-	-	-	X	
*OASL/TCF1*	rs7957197	-	-	-	-	-	-	-	-	-	-	-	-	-	-	-	-	-	-	X	
*PRC1*	rs8042680	-	-	-	-	-	-	-	-	-	-	-	-	-	-	-	-	-	-	X	
*RBMS1/ITGB6*	rs7593730	-	-	-	-	-	-	-	-	-	-	-	-	-	-	-	-	-	-	X	
*TLE4*	rs13292136	-	-	-	-	-	-	-	-	-	-	-	-	-	-	-	-	-	-	X	
*TP53INP1*	rs896854	-	-	-	-	-	-	-	-	-	-	-	-	-	-	-	-	-	-	X	
*ZBED3*	rs4457053	-	-	-	-	-	-	-	-	-	-	-	-	-	-	-	-	-	-	X	
*ZFAND6*	rs11634397	-	-	-	-	-	-	-	-	-	-	-	-	-	-	-	-	-	-	X	

**Legend Table 4**: SNPs, single nucleotide polymorphisms. ^1^Another variant in perfect linkage disequilibrium (R^2^ > 0.90) was used; ^2^This variant is in high LD (R^2^ ≤ 0.90 and > 0.60) with the reference variant; ^3^This variant is in low (R^2^ ≤ 0.60 and > 0.05) with the reference variant; ^4^This variant has an R^2^ ≤ 0.05 with the reference variant. The first column lists all SNPs included in genetic tests for T2D, either used by DTC companies or available from published studies. “X” denotes that the SNP was included in the genetic risk model and “-” denotes that the SNP was not included.

Another important aspect when testing the performance of a prediction model is the model calibration. Measures of calibration were presented in some of the T2D risk prediction studies and generally showed sufficient model fit.[36] [37] [38] [39] [41] [43] [45] [46]

***Clinical Utility*:**Net benefit of test in improving health outcomes.

We assessed clinical utility as the added benefit of the test beyond traditional clinical predictors in improving health outcomes, and as the impact of genetic testing on attitudes, beliefs and health related behavior in individuals who receive genetic risk information.

First, clinical utility is reflected in the impact of a risk prediction model on the classification of individuals in risk groups for which the preventive interventions differ. Percentage of reclassification and the net reclassification improvement (NRI) are recently developed measures that assess this aspect of clinical utility. Reclassification is the percentage of individuals that change from one risk category based on the original prediction model to a different risk category based on the updated model. NRI separately considers the reclassification in cases and non-cases. Cases are correctly classified when they move to a higher risk category and wrongly classified when they move to a lower category. Non-cases move correctly to a lower category and wrongly to a higher. NRI is the sum of the net correct moves: the proportion of cases moving up minus the proportion of cases moving down, plus the proportion of non-cases moving down minus the proportion of non-cases moving up.[49] **Table 5** shows the amount of reclassification resulted from the addition of genetic information to clinical data in T2D risk prediction, either directly reported in the original studies or calculated from reclassification tables available from original papers.[23] Since most genetic risk prediction studies in T2D have been performed in European populations (see **Table 3**) it is impossible to generalize the performance of the genetic tests to populations with different ancestry. Furthermore, the incidence rates of T2D vary even within European ancestry groups. As a result, no clinically defined risk categories exist that can be applied across different populations where the underlying risk of T2D varies and, therefore, the cut-off values chosen to define the risk groups differ among studies. This is an important aspect in the interpretation of reclassification measures, as the choice of cut-off has a high impact on the percentage of reclassification observed.[23] In consequence, the assessment of NRI in the absence of clinically estimated cut-offs is of limited value.

**Table 5.** Reclassification measures from genetic risk prediction studies in type 2 diabetes

**Table:** 

**Study reference**	**Updated model**	**Cut-off values **	**NRI, % (P value)**	**Reclassification, %**
Meigs et al.[23] [37]	Most complete clinical model plus 18 SNPs	2 and 8%	2.13 (0.17)	6.28
Lyssenko et al.[23] [36] 1)Malmö study 2)Botnia study	Most complete clinical model plus 11 SNPs	10 and 20%	1) 4.5 (2.5x10^-5^) 2) 8.79 (0.13)	1) 15.59 2) 9.44
Talmud et al.[43]	1) Cambridge risk score plus 20 SNPs 2) Framingham risk score plus 20 SNPs	5, 10 and 15%	1) 4.6 (0.17) 2) -3.2 (0.35)	1) 16 2) 12.3
de Miguel-Yanes et al.[46]	Most complete clinical model plus 40 SNPs	2 and 8%	1) < 50 yrs: 10.2 (0.001) 2) ≥ 50 yrs: 0.4 (0.7) 3) All: 1.8 (0.2)	NA

Legend **Table 5**: NA, not assessed.

Second, when the impact on outcome prediction is not available, clinical utility is reflected in the public interest and health care provider interest in genetic testing, the uptake of the tests and the effect of testing on outcomes such as adherence to lifestyle changes or to medication for prevention and treatment of disease.

A survey conducted among primary care physicians and endocrinologists (n = 304) and patients (152 non-diabetic and 89 with T2D) assessed beliefs regarding the clinical use of genetic testing for T2D. Subjects answered questions related to three domains: testing for risk prediction, testing to motivate behavior change and testing to guide medication prescription. Most physicians (88%) and patients (79%) were in favor of genetic testing in general. However, patients were more likely than physicians to request genetic testing for risk prediction and treatment guidance. Patients, and to a lesser extent physicians, expressed expectations that knowledge of genetic risk would motivate adoption of preventive lifestyle recommendations and increase adherence to treatment.[50]

We identified four registered clinical trials (see **Links** for search strategy) that aim to assess the impact of genetic testing on risk perception and behavior change in patients with T2D: 

o   **Genetic Counseling and Lifestyle Change for Diabetes Prevention (GC/LC):** “This study will examine the impact of diabetes genetic counseling on patient motivation and disease prevention behaviors among subjects with pre-diabetes. Intervention subjects will be provided with their individual diabetes genotype risk score derived from aggregating the combined results of 37 diabetes risk-associated genetic loci. Controls will not be tested. All subjects will be enrolled in a 12-week diabetes prevention program.” (ClinicalTrials.gov identifier: NCT01034319)

o   **The Impact of Genetic Testing for Type 2 Diabetes on Health Behaviors: **“We will evaluate the impact of genetic testing for type 2 diabetes on psychological, health behavior, and clinical outcomes.” (ClinicalTrials.gov Identifier NCT01060540) The genetic test consists of SNPs in the *TCF7L2*, *PPARG* and *KCNJ11* genes.

o   **Effect of Type 2 Diabetes Genetic Risk Information on Health Behaviors and Outcomes (TDE): **“The primary objective of the study is to assess the clinical utility of a genetic test for Type 2 diabetes risk in combination with standardized risk assessment compared with standardized risk assessment alone.” (ClinicalTrials.gov Identifier NCT00849563) Variants not specified.

o   **Predictive Genetic Risk Assessment Trial (PGT): **“A critical goal of this clinical trial is to understand how individual patients and their doctors perceive and respond to genetic risk information that is largely uncertain.” (ClinicalTrials.gov Identifier NCT00782366) Variants not specified.

**Methods**

To identify published reviews, recommendations and guidelines on genetic testing for T2D risk prediction we searched: the Agency for Healthcare Research and Quality (AHRQ), the Cochrane Collaboration, the US Preventive Task Force, the Evaluation of Genomic Applications in Practice and Prevention Working Group, the National Institute for Health and Clinical Excellence, the NHS Evidence - National Library of Guidelines; the Canadian Medical Association Infobase: Clinical Practice Guidelines, the European Society for Human Genetics. To retrieve information about companies that offer DTC genetic testing for T2D risk prediction we performed a search in Google, followed the list of companies from a published review on DTC genomic companies^27^ and collected additional information from discussions with other researchers.

**Links**

·       **PubMed:** type 2 diabetes AND (genetic markers OR risk polymorphisms OR genetic score* OR susceptibility variants OR genetic risk factors OR genetic testing OR genotype score) AND (risk assessment OR disease prediction OR risk prediction OR discriminative value OR ROC curve)

·       **HuGE Navigator:** type 2 diabetes[Text+MeSH]>>Gene-gene interactions, Genetic testing[Category] 

·       **ClinicalTrials.gov:** type 2 diabetes AND genetic tests | type 2 diabetes

·       **U.S.** **Food and Drug Administration:** No information indentified

Last updated: 21 December, 2010

**Acknowledgements**

We would like to thank Dr. Heidi Howard from the K.U.Leuven Centre for Biomedical Ethics and Law, Leuven, Belgium, for providing assistance in the identification of commercial DTC genomic companies that offer testing for T2D risk.

**Funding information**

This study was supported by the Centre for Medical Systems Biology (CMSB) in the framework of the Netherlands Genomics Initiative (NGI). Furthermore, this project was sponsored by the VIDI grant of the Netherlands Organization for Scientific Research (NWO). 

Dr. Meigs is supported by NIDDK R01 DK078616 and NIDDK K24 DK080140.

**Competing interests**

Dr. Meigs serves on an advisory panel for Interleukin Genetics, Inc. 
